# Reverse vascular pedicle digital island flap with preservation of the dorsal branch of the digital nerve

**DOI:** 10.1080/23320885.2019.1632202

**Published:** 2019-06-22

**Authors:** Takeo Osaki, Tadashi Nomura, Kazunobu Hashikawa, Hiroto Terashi

**Affiliations:** Department of Plastic Surgery, Kobe University Graduate School of Medicine, Kobe, Hyogo, Japan

**Keywords:** Reverse vascular pedicle digital island flap, fingertip amputation, dorsal branch of the digital nerve, fingertip reconstruction

## Abstract

A reverse vascular pedicle digital island flap is a useful treatment option for reconstruction in fingertip amputation. We describe a surgical procedure to preserve the dorsal branch of the digital nerve in the middle phalanx during elevation of this flap with favourable outcomes.

## Introduction

In fingertip amputation, when replantation or composite grafting is difficult, flap reconstruction, revision amputation, and open treatment are the treatment options, and the treatment is selected depending on the wound condition and the patient’s desire (the treatment period should be short or the finger length should be kept as long as possible). In flap reconstruction, when the defect is small, oblique triangular flaps [1] or palmar advancement flaps [2,3] are used. When the defect is large, reverse vascular pedicle digital island flaps [4], reverse island flaps based on the dorsal branch of the digital artery [5], cross finger flaps [6], and thenar flaps [7,8] are used, among other local options. Among these, reverse vascular pedicle digital island flaps have a relatively good prognosis with respect to sensory recovery [9,10]. These flaps can be used in a single stage, whereas two stages are required for cross-finger or thenar flaps.

In this study, we performed a surgical procedure to preserve the dorsal branch of the digital nerve in the middle phalanx when elevating the reverse vascular pedicle digital island flap. To our knowledge, there are no reports of this nerve branch being preserved when elevating this flap. We also discuss the advantages and disadvantages of the procedure.

## Case report

**Figure 1.**
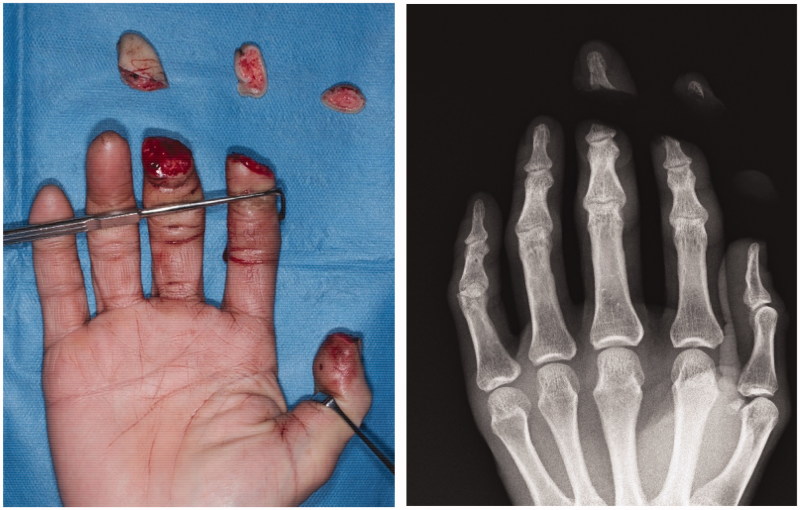
Fingertip amputation of the right thumb, index finger, and middle finger.

A 50-year-old man experienced amputation of the thumb, index finger, and middle finger of the right hand in a rubber cutting machine in a work-related accident and was brought to our hospital as an emergency. The thumb and index fingers were completely amputated at the position of zone I [11,12] and the middle finger was amputated at zone II (Figure 1). He was a right-handed field worker who cut rubber. We used a composite graft for the thumb and the index finger. Replantation of the middle finger was abandoned due to severe damage, and reconstruction with a reverse vascular pedicle digital island flap was performed on the day of injury because the bone was exposed at the wound and he wanted to keep the finger length as long as possible.

**Figure 2.**
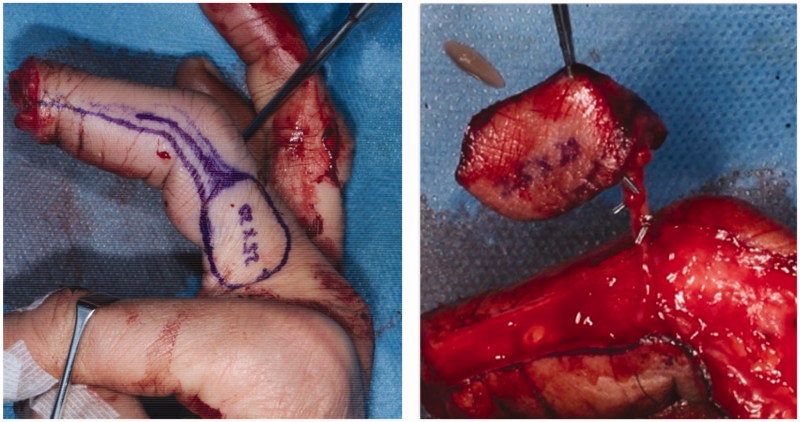
A flap of 25 × 20 mm was made on the radial side at the base of the proximal phalanx of the right middle finger.

**Figure 3.**
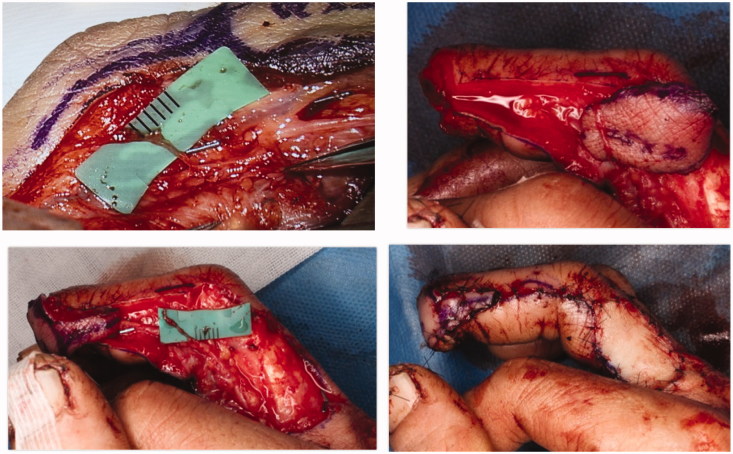
Since the dorsal branch of the digital nerve was identified in the middle phalanx, the flap was passed under the nerve to preserve it.

Surgery was performed under general anaesthesia. All surgical steps were performed under a microscope. A flap of 25 × 20 mm was made on the radial side at the base of the proximal phalanx of the right middle finger (Figure 2), the vascular pedicle was ligated proximally after clamping to confirm finger blood circulation and isolated from the digital nerve, and the flap was elevated. At the same time, the dorsal branch of the digital nerve was identified in the middle phalanx, and the flap was passed under the nerve to preserve the nerve (Figure 3). The fingertip defect was covered with the flap, and the donor site was closed with a skin graft. The flap survived without any venous congestion.

**Figure 4.**
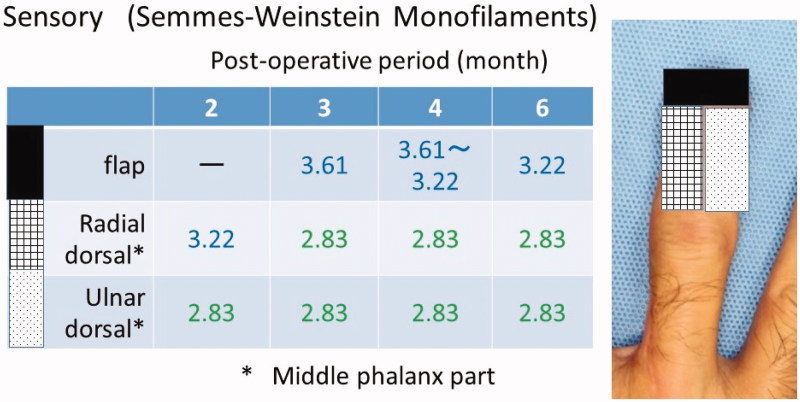
Sensory (Semmes-Weinstein Monofilaments).

**Figure 5.**
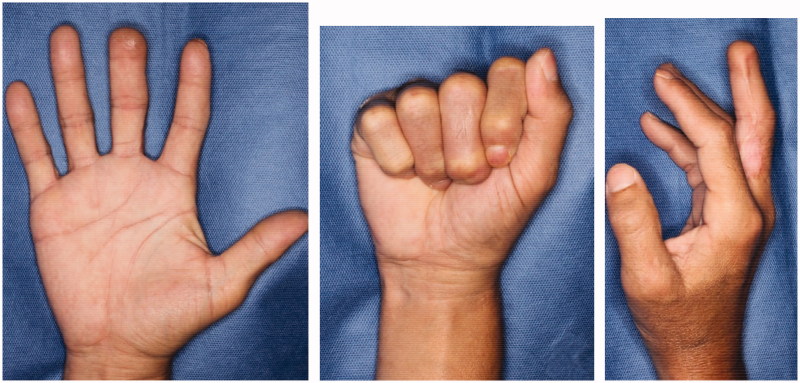
At half a year after the operation, there is no limitation on the range of motion, and the contour is also good.

The postoperative sensory recoveries of the flap and the dorsal skin of the distal phalanx were excellent. In the Semmes–Weinstein monofilament test, the sensation of the flap returned to 3.22 (blue) and the dorsal sensation of the radial side recovered to 2.83 (green) early after surgery (Figure 4). At half a year after the operation, there was no limitation in the range of motion, and the contour was good (Figure 5).

## Discussion

A method to reconstruct the fingertip using a retrograde digital artery flap has been reported by Lai et al. as a reverse digital artery flap [13] and by Kojima et al. as a reverse vascular pedicle digital island flap [4]. Since then, its usefulness has been reported numerous times, and it has been widely used for reconstruction of the fingertip [9,10,14,15]. The sensation of the finger is controlled by the digital nerve and the digital dorsal nerve, and it varies from individual to individual. Hayashi et al. [16] examined the courses of the dorsal branch of the digital nerve and the dorsal digital nerve of 80 fingers. They found that the dorsal branch of the digital nerve often branches at the proximal phalanx, but a branching type also occurs at the middle phalanx. It was also reported that the proportions of nerve branches differ depending on the finger (0% for the index finger, 30% for the middle finger, 10% for the ring finger, and 5% for the little finger). During elevation of the reverse vascular pedicle digital island flap, if the dorsal branch of the digital nerve is present in the middle phalanx, its branch is usually cut. In such cases, the sensation of the dorsum of the distal phalanx may decline. In our case, by passing the flap under the dorsal branch of the digital nerve, the branch was preserved. After surgery, the sensation of the dorsal distal region temporarily declined. This appeared to be due to neurapraxia caused by touching, even when the nerve was not cut. However, the sensation recovered quickly, and this effect may be attributed to the preservation of the nerve.

In addition, sensory recovery in insensate flaps occurs due to collaterals sprouting from adjacent intact nerves [17]. The dorsal distal region is the tissue around the flap. In this case, the sensation of the flap recovered well. Preserving the dorsal distal sensation could also contribute to the recovery of the sensation of the reverse vascular pedicle digital island flap.

However, this method has a disadvantage. The dorsal branch of the digital nerve is thin and complicated to peel off; hence, the surgery time could be prolonged. Moreover, the reverse vascular pedicle digital island flap tends to cause congestion [9,18,19]. Therefore, a method has been developed to elevate the flap with a skin pedicle to prevent congestion [20]. However, for preserving the dorsal branch of the digital nerve, it is impossible to create the skin pedicle because it should be only a vascular pedicle.

The dorsal branch of the digital nerve is generally considered unimportant owing to the presence of the contralateral nerve. For further assessment of the significance of this surgical procedure, a larger series with a control group is required.

## Conclusion

In this report, we described a procedure for reconstruction of the fingertip with a reverse vascular pedicle digital island flap, which preserved the dorsal branch of the digital nerve. The sensory recoveries of the flap and the dorsal distal phalanx were favourable. As this is a case report, we cannot describe the effectiveness currently; however, we consider that the method of elevation may be useful.
